# Retropharyngeal emphysema following adenoidectomy: a case report and review of literature

**DOI:** 10.1093/jscr/rjag791

**Published:** 2026-09-09

**Authors:** Zahraa Jumah Almuhanna, Rabab Hussain Alzanadi, Hussain Abdullah Almulla

**Affiliations:** ENT Department, King Fahad University Hospital, Khobar, Saudi Arabia; ENT Department, King Fahad University Hospital, Khobar, Saudi Arabia; Pediatric ENT Department, Easterm Health Cluster, Ali bin Abi Talib St, Al Muraikabat, Dammam 32253, Saudi Arabia

**Keywords:** retropharyngeal cellulitis, retropharyngeal emphysema, retropharyngeal abscess, deep neck space abscess, adenoidectomy, case report

## Abstract

Retropharyngeal infection and emphysema are rare clinical entities, with only eight reported cases in the literature. One of the uncommon risk factors is a recent history of adenotonsillectomy, which may be associated with an increased risk of retropharyngeal infection. Thereby, we report a case of a 2-year-old female who presented with a high-grade fever and neck stiffness following adenotonsillectomy. Neck X-ray and computed tomography scan of the neck confirmed the diagnosis of retropharyngeal infection with emphysema. The patient was effectively managed with an intravenous course of dual broad-spectrum antibiotics. Early identification and prompt management are essential to avoid serious complications. The current case demonstrates a rare complication with a delayed onset compared to the previously reported cases, and it further emphasizes the importance of the high index of clinical suspicion to diagnose and promptly manage similar cases.

## Introduction

Adenoidectomy remains one of the most common procedures in otolaryngology. It is considered a safe procedure with low complication rates; although rare, complications may include hemorrhage, velopharyngeal insufficiency, and nasopharyngeal stenosis [1–3]. One of the rare complications is retropharyngeal infection or emphysema, which was first reported in 1995 and has been reported only seldom since [4, 5]. The retropharyngeal space is a potential space spanning from the skull base to the posterior mediastinum; it contains lymph nodes that drain multiple head and neck areas, including the nasopharynx [6, 7]. Retropharyngeal infection is more common in pediatrics, symptoms include fever, sore throat, and restricted neck mobility. The principle management includes intravenous antibiotics, while surgical drainage is reserved for severe cases with well-formed abscesses [5–8]. The purpose of this study is to report a case of retropharyngeal infection and emphysema following an adenoidectomy.

## Case presentations

**Figure 1:**
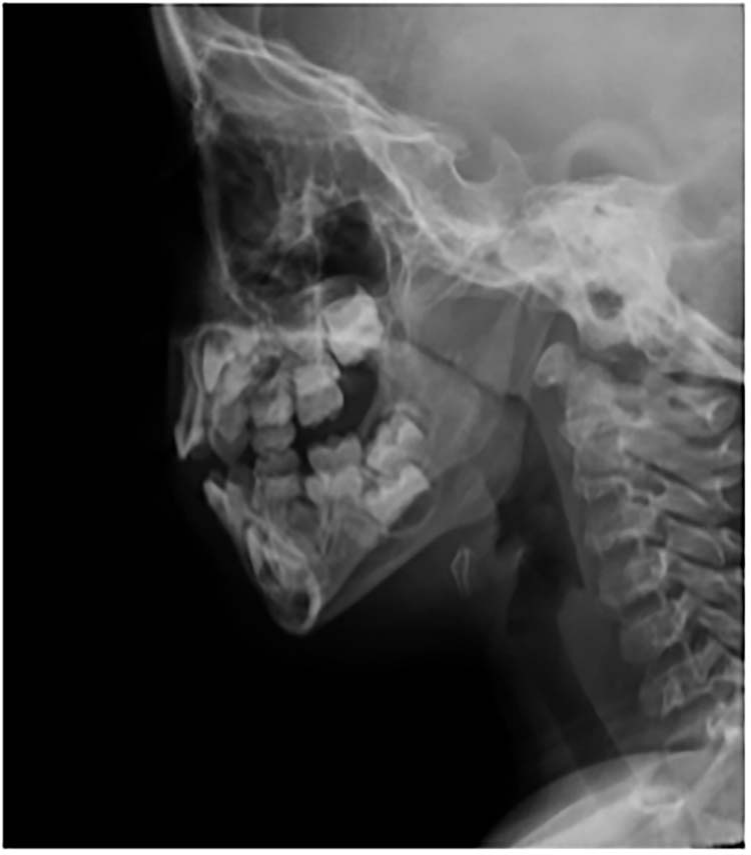
Preoperative lateral soft tissue neck X-ray showing enlarged adenoidal tissue nearly obstructing the nasopharynx.

**Figure 2:**
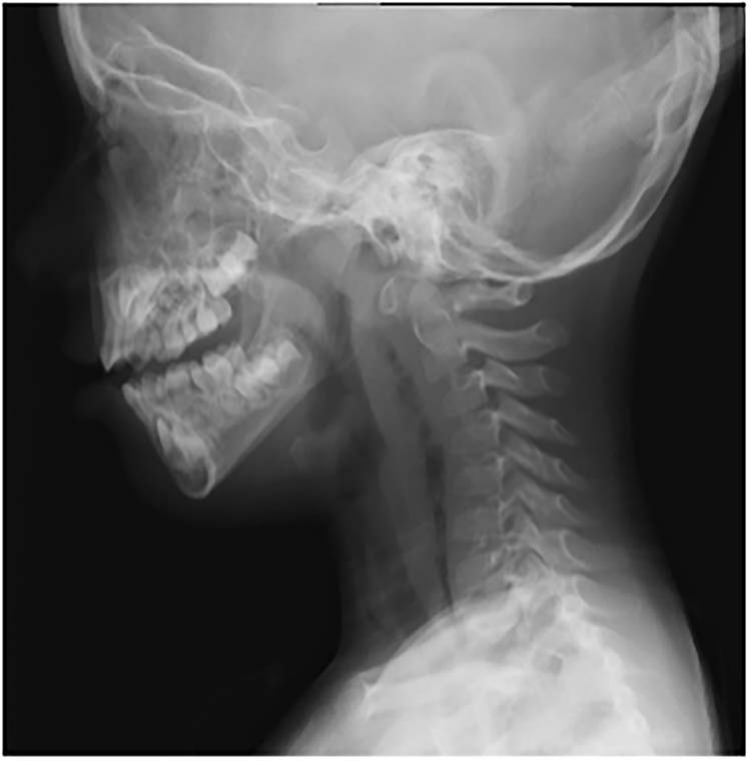
Postoperative lateral soft tissue neck X-ray showing thickened retropharyngeal area with air foci.

**Figure 3:**
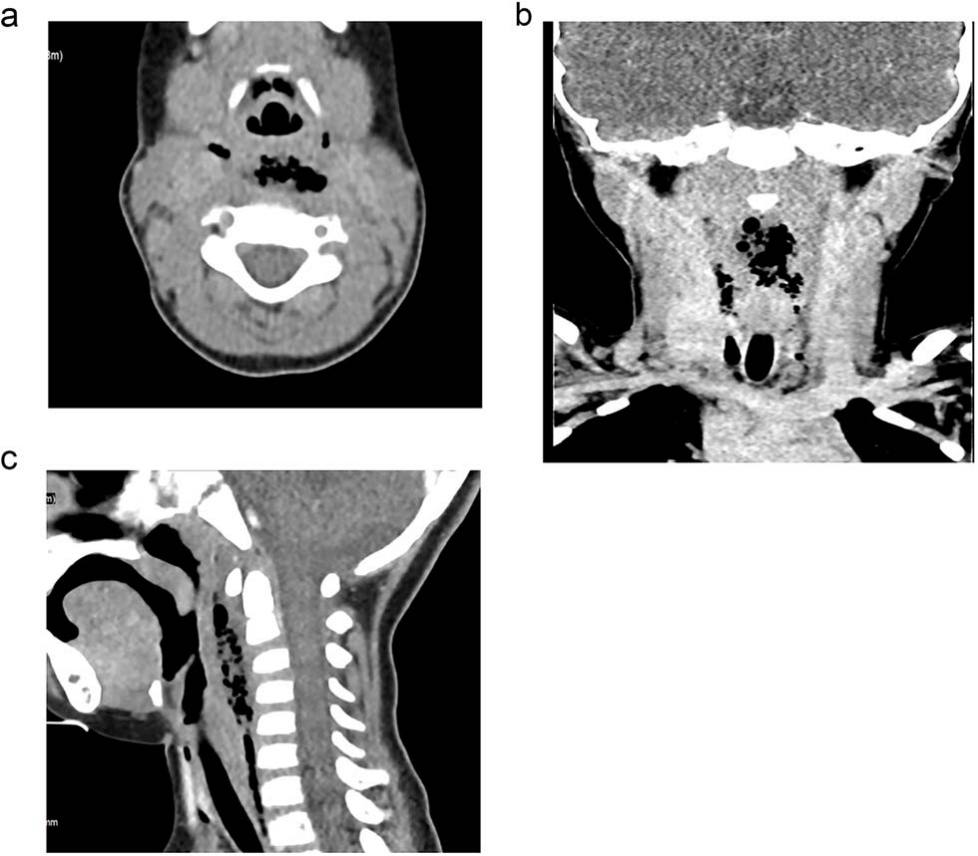
Coronal (a), axial (b), and sagittal (c) CECT scan images showing thickening of the retropharyngeal space with diffuse, multi-lobulated gas densities, measuring ~9.75 × 1.42 × 2.4 cm in its maximum craniocaudal, anteroposterior, and transverse dimensions, respectively; the gas/air locules are seen tracking anteriorly along the paraglottic perilaryngeal space and anterior neck; faint peripheral enhancement is noted around the multi-loculated gas densities after IV contrast administration.

**Figure 4:**
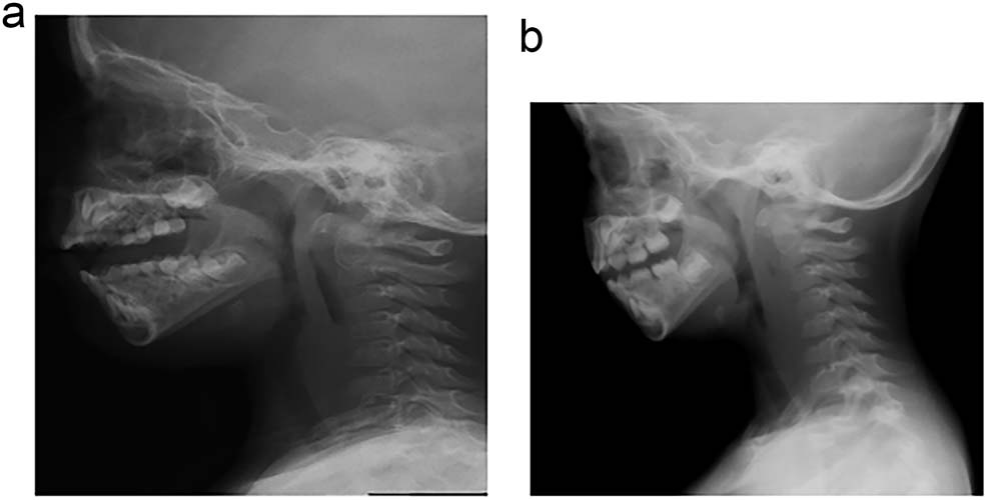
Lateral soft tissue neck X-rays at Day 5 (a) and Day 9 (b) of intravenous antibiotic course showing progressive resolution of retropharyngeal air foci.

A 2-year-old previously healthy girl presented to the otorhinolaryngology clinic complaining of snoring and mouth breathing. Lateral neck X-ray showed adenoid hypertrophy (Fig. 1). Nasal sprays were unsuccessful; therefore, she underwent an adenotonsillectomy. Intraoperative examination revealed a large adenoid. The adenoidectomy was primarily done using an adenoid curette under direct vision with a mirror, followed by suction monopolar for remnants. The procedure went smoothly with an uneventful postoperative course; she was discharged the following day in a stable condition. During the 2-week postoperative follow-up, her family reported a stable recovery. However, she was brought to the emergency department 1 week later with a 1-day history of high-grade fever and neck stiffness. Her symptoms were preceded by a dry cough, no other airway symptoms were reported. Physical examination revealed a high-grade fever, she was otherwise vitally stable with no respiratory distress signs. Thick postnasal discharge was noted in throat examination, along with limited neck mobility. Flexible nasolaryngoscopy was unremarkable apart from colored nasopharyngeal discharge. Laboratory work-up revealed leukocytosis (29.4) and high inflammatory markers (C-reactive protein of 149.4 and erythrocyte sedimentation rate of 70); blood cultures were negative. Lateral neck X-ray revealed a thickened retropharyngeal space with evident air (Fig. 2). Contrast-enhanced computed tomography (CECT) of the neck further demonstrated retropharyngeal multi-loculated densities which could represent retropharyngeal cellulitis with early phlegmon and emphysema (Fig. 3). The patient was admitted and was started on dual broad-spectrum intravenous antibiotics (vancomycin and piperacillin/tazobactam), surgical management was reserved in case of clinical deterioration as the CT did not illustrate a loculated abscess, with only a faint enhancement around the gas densities representing cellulitis and possible early phlegmon. Close monitoring with involvement of the pediatric infectious disease team was decided. The patient showed significant clinical and radiological improvement with multiple interval neck X-rays (Fig. 4). She was discharged 10 days after admission in a stable condition on a high-dose oral amoxicillin/clavulanic acid, and remained asymptomatic during her regular follow-up.

## Discussion

Retropharyngeal abscess and/or emphysema postadenoidectomy are rare entities with only eight reported cases in the literature. Typically, the onset is in the first 2 weeks during the postoperative course, as documented in multiple previous reports. However, two articles reported cases that developed symptoms as early as during the post-extubation period and 20 min after transfer to the postoperative recovery area [9–12]. Compared with previous reports, our case had the most delayed onset, occurring 3 weeks postoperatively, highlighting the importance of follow-up and red-flags instructions upon discharge. A recent history of sore throat or tonsillitis prior to the surgery has been reported, which might predispose to the development of retropharyngeal complications; however, no recent preoperative infection was documented in the current case [5, 12]. Moreover, a study conducted in 2008 found that 13.9% of children who were diagnosed with retropharyngeal or parapharyngeal abscess had a history of prior adenoidectomy. It also reported that tonsillectomy and adenoidectomy were found to transiently decrease immunoglobulin (Ig) levels, namely IgM, IgG, and IgA, increasing the susceptibility to develop pharyngeal infections [8]. Another predisposing factor can be related to the surgical trauma inflicted on the posterior pharyngeal wall, creating a bacterial entry port. This breach acts as a one-way valve trapping air and saliva, subsequently leading to bacterial overgrowth and ultimately cellulitis, phlegmon, and abscess [12]. Interestingly, multiple studies proposed that postoperative vomiting may predispose to increased pressure over the oropharynx, thus reinforcing air into the soft tissue [1, 13]. Retropharyngeal infections frequently present with fever, neck pain/stiffness, and dysphagia. Nonetheless, symptoms sometimes may be nonspecific, leading to delayed diagnosis [12]. Our patient presented with toxic general appearance, fever, and restricted painful neck mobility. The risks of overlooking retropharyngeal infections are demonstrated in the case published by Holm-Hansen *et al*., who reported a patient initially presenting with dyspnea, chest and back pain, and unremarkable X-ray. The patient was investigated for aspiration pneumonia and meningitis until imaging with CT scan established the diagnosis of retropharyngeal abscess [13]. Imaging is crucial to confirm and localize deep neck space infections. Lateral neck radiographs are the initial modality, and might reveal a widened retropharyngeal area. Contrast-enhanced CT scan is the gold standard to diagnose and evaluate deep neck space infections and to plan surgical drainage if needed. Management of retropharyngeal infections entails conservative and/or surgical management; the decision should be tailored individually for each patient [12]. Conservative management can be effective if the clinical presentation allows, as reported in multiple cases. However, if surgical drainage was decided, the transoral approach is the principal approach for retropharyngeal space [4, 5, 9, 12, 13]. The decision to proceed with conservative management in this case was made after clinical and radiological assessment. The provisional diagnosis of retropharyngeal cellulitis and early phlegmon with no well-loculated abscess allowed the trial of intravenous antibiotics and close monitoring, which proved effective.

## Conclusion

Although adenoidectomy is considered a safe procedure, complications can arise. We present a rare case of retropharyngeal cellulitis and emphysema following adenoidectomy. Clinical presentation with radiological imaging was crucial to reach the diagnosis. Clinicians should maintain a high index of suspicion. Early initiation of treatment with multidisciplinary team involvement ensures effective management.
